# Bot Gummosis of Lemon (*Citrus* × *limon*) Caused by *Neofusicoccum parvum*

**DOI:** 10.3390/jof7040294

**Published:** 2021-04-14

**Authors:** Francesco Aloi, Mario Riolo, Rossana Parlascino, Antonella Pane, Santa Olga Cacciola

**Affiliations:** 1 Department of Agriculture, Food and Environment, University of Catania, 95123 Catania, Italy; francesco.aloi@unipa.it (F.A.); mario.riolo@unirc.it (M.R.); rossana.parlascino@gmail.com (R.P.); 2 Department of Agricultural, Food and Forest Sciences, University of Palermo, 90128 Palermo, Italy; 3 Council for Agricultural Research and Agricultural Economy Analysis, Research Centre for Olive, Citrus and Tree Fruit-Rende CS (CREA-OFA), 87036 Rende, Italy; 4 Department of Agricultural Science, Mediterranean University of Reggio Calabria, 89122 Reggio Calabria, Italy

**Keywords:** Botryosphaeriaceae, *ITS*, *TEF1*, *TUB2*, citrus, trunk and branch cankers, pathogenicity, Italy

## Abstract

*Neofusicoccum parvum*, in the family Botryosphaeriaceae, was identified as the causal agent of bot gummosis of lemon (*Citrus* × *limon*) trees, in the two major lemon-producing regions in Italy. Gummy cankers on trunk and scaffold branches of mature trees were the most typical disease symptoms. *Neofusicoccum parvum* was the sole fungus constantly and consistently isolated from the canker bark of symptomatic lemon trees. It was identified on the basis of morphological characters and the phylogenetic analysis of three loci, i.e., the internal transcribed spacer of nuclear ribosomal DNA (ITS) as well as the translation elongation factor 1-alpha (*TEF1*) and β-tubulin (*TUB2*) genes. The pathogenicity of *N. parvum* was demonstrated by wound inoculating two lemon cultivars, ‘Femminello 2kr’ and ‘Monachello’, as well as citrange (*C. sinensis* × *Poncirus trifoliata*) ‘Carrizo’ rootstock. In artificial inoculations, the fungus was very aggressive on lemons and weakly virulent on citrange, consistently with symptoms observed in the field as a consequence of natural infections. This is the first report of *N. parvum*, both in a wide and in a strict taxonomic sense, as a pathogen of lemon in Italy.

## 1. Introduction

Trunk and branch cankers caused by species of Botryosphaeriaceae on citrus were traditionally referred to as Diplodia gummosis and Dothiorella gummosis [1,2]. More recently the comprehensive term bot gummosis was introduced to indicate trunk and branch cankers of citrus caused by fungi in the Botryosphaeriaceae family in California [3]. All common names of the disease refer to the most typical symptom, i.e., the gum exudate oozing from the bark cankers. Another typical symptom of the disease is a chocolate brown to dark brown discoloration of the wood which becomes evident after peeling the bark. Originally, the fungi causing Diplodia gummosis and Dothiorella gummosis were identified as *Diplodia natalensis* and *Dothiorella gregaria* (syn. *Botryosphaeria ribis*), respectively [1]. However, identifications of Botryosphaeriaceae species prior to the application of taxonomic criteria based on DNA sequencing and phylogenetic inference should be re-examined carefully as the taxonomy of this family of fungi has substantially changed during the last decade and is still evolving rapidly. As a matter of fact, very recently new species have been described and species that were previously separated on the basis of both multi-loci phylogenetic analysis and morphological characters have been reduced to synonymy on the basis of same genetic markers previously regarded as discriminatory [4,5,6,7]. After the introduction of modern molecular taxonomy, several Botryosphaeriaceae species were reported to be associated to bot gummosis in California, including *Diplodia mutila*, *D. seriata*, *Dothiorella iberica* (very recently indicated as a possible synonim of *Do. sarmentorum*), *Spencermartinsia viticola* (syn. *Do. viticola*), *Lasiodiplodia parva*, *Neofusicoccum australe*, *N. luteum*, *N. mediterraneum*, *N. parvum* and *Neoscytalidium dimidiatum* (syn. *Ne. hyalinum*, formerly *Hendersonula toruloidea*) [3,8,9]. *Neofusicoccum parvum* and *Diaporthe foeniculina* were reported to be responsible for shoot blight and branch cankers of citrus in Greece, but in pathogenicity tests *N. parvum* was significantly more virulent than *D. foeniculina* [10]. *Dothiorella viticola* was identified as the causal agent of bot gummosis of citrus in Tunisia [11]. Moreover, symptoms of bot gummosis were reproduced by artificially inoculating citrus trees with *Neofusicoccum batangarum* (very recently reduced to synonymy with *N. ribis*), the causal agent of a destructive disease of cactus pear in minor islands of Sicily [12,13]. In the island of Malta, about 80 marine miles south of Sicily, symptoms very similar to bot gummosis were reported on lemon (*Citrus* × *limon*) trees to be caused by species of *Diaporthe* [14].

During recent surveys in citrus growing areas in Sicily and Calabria, the two major citrus producer regions of Italy, symptoms of bot gummosis were observed to be common on mature, fruit-bearing lemon trees. This study was aimed at determining the etiology and the distribution of the disease in the major producing areas of lemon in Italy.

## 2. Materials and Methods

### 2.1. Fungal Collection and Isolation

**Figure 1:**
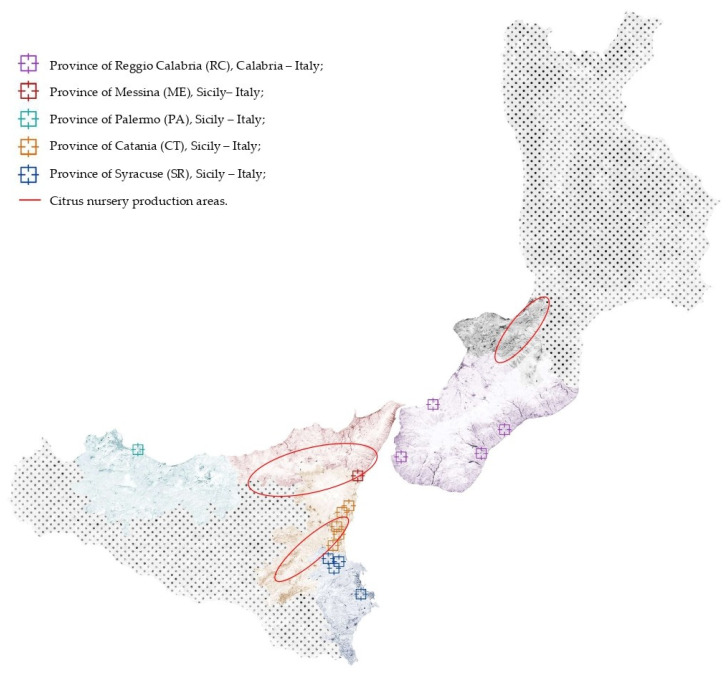
Map of southern Italy, including Sicily and Calabria, where sampling sites are indicated with colored squares (each color corresponds to a different province) while major citrus nursery production areas are indicated with circles.

Five citrus-growing areas in southern Italy, four in Siciliy, including the province of Palermo and the three Sicilian districts in the provinces of Catania, Messina, and Syracuse to which the Protected Geographical Indication trademark was granted by EU, and one in Calabria (province of Reggio Calabria), were surveyed in 2017 and 2018 (Figure 1). The geographical map in Figure 1 also shows the close proximity between the surveyed areas and the major districts of Calabria and Sicily where citrus nursery plants production is concentrated.

Approximately 15 to 20 bark pieces from trunk and branch cankers were collected from 25 randomly selected symptomatic trees (approximately 5 to 60-yr-old). Samples were transported in a cooler to the laboratory of Molecular Plant Pathology at the Department of Agriculture, Food, and Environment (Di3A) of the University of Catania, Italy.

Bark pieces with the typical brown discoloration of the wood were rinsed with deionized water to remove organic debris, blotted dry with paper towels, then briefly flamed after dipping in 95% ethanol for 3 s. Small sections (approximately 2 to 3 mm) from the inner face of necrotic bark were cut with a sterile scalpel and placed in Petri dishes onto potato dextrose agar (PDA; Difco Laboratories, Detroit, MI, USA) amended with 100 µg/ml streptomycin (Sigma-Aldrich, Darmstadt, Germany). Cultures were incubated in the dark for 4 days at 25 °C.

Pure cultures of fungal isolates were obtained by monoconidial cultures. The list of isolates characterized in this study is reported in Table 1.

### 2.2. Morphological Characteristics and Cardinal Temperatures for Growth of the Isolates

The isolates were induced to sporulate by plating them on PDA containing sterilized pine needles [4], and incubating at room temperature (ca. 20 to 25 °C) under diffused day light or near-UV light, until pycnidia developed. For microscopy, pycnidia and conidia were mounted in sterile distilled water or 100% lactic acid and observed microscopically at ×40 and ×100 magnifications, with an Axioskop (Zeiss) microscope. Images were captured with an AxioCam MRc5 camera (Zeiss), and measurements were made with the software AxioVision. For each isolate, 50 conidia were randomly selected, and their lengths, widths, and shape were recorded. For the dimension of pycnidia, 20 measurements were made. Colony characters and pigment production were noted after 4 to 6 d of growth on PDA or malt extract agar (MEA) at 25 °C, in the dark.

Colony colors (upper and lower surfaces) were rated and the conidial characteristics observed were compared with those that were reported in previous studies [15,16,17,18,19].

Radial growth rate and cardinal temperatures for radial growth were determined by growing the isolates on PDA in Petri dishes (9 cm diam.), and incubating them at 5, 10, 15, 20, 25, 30, and 35 °C, in the dark. Means of radial growth at the different temperatures were adjusted to a regression curve using Statgraphics Plus 5.1 software (Statgraphics Technologies Inc., The Plains, VA, USA), and the best polynomial model was chosen based on parameter significance (*p* < 0.05) and coefficient of determination (R2) to estimate the optimum growth temperature for each isolate. Four replicates of each isolate were evaluated and each experiment was repeated twice.

### 2.3. Amplification and Sequencing

Genomic DNA was isolated from 1-week-old cultures grown on PDA at 25 °C in the dark using the procedure of Schena and Cooke [20]. The internal transcribed spacer (ITS) region of the ribosomal DNA was amplified and sequenced with primers ITS1/ITS4 [21], part of the translation elongation factor 1 alpha gene (tef1) was sequenced and amplified with primers EF1-728F/EF1-986R [22], and the β-tubulin gene (tub2) was sequenced and amplified with Bt2a and Bt2b [23].

The PCR amplifications were performed on a GeneAmp PCR System 9700 (Applied Biosystems, Monza-Brianza, Italy). All PCRs were performed by using the Taq DNA polimerase recombinant (Invitrogen™, Carlsbad, 254 CA, USA) and carried out in a total volume of 25 μL containing the following: PCR Buffer (1X), dNTP mix (0.2 mM), MgCl_2_ (1.5 mM), forward and reverse primers (0.5 μM each), Taq DNA Polymerase (1 U), and 1 μL of genomic DNA.

The reaction protocol for ITS and β-tubulin included an initial preheat at 94 °C for 2 min; followed by 35 cycles of denaturation at 94 °C for 15 s, annealing at 58 °C for 15 s, and extension at 72 °C for 45 s; and final extension at 72 °C for 10 min. The translation EF α-1 included an initial denaturation at 95 °C for 8 min; followed by 35 cycles of 95, 58, and 72 °C for 15, 20, and 60 s, respectively; and a final extension at 72 °C for 10 min. The amplicons were detected in 1% agarose gel and purified products were sequenced by Macrogen Europe (Amsterdam, The Netherlands). Sequences were analyzed by using FinchTV v.1.4.0 [24] and MEGA7 [25] and the consensus sequences were deposited in Genbank.

### 2.4. Molecular Identification and Phylogenetic Analyses

Sequences of isolates were associated to a species by the application of BLAST algorithm on the NCBI nucleotide database. Species identity was confirmed by a multi-loci phylogenetical analysis including sequences from taxa that best matched the blast species identity [26,27,28]. The list of isolates used as reference is reported in Table 2. * Netto et al., unpublished. 

For all isolates, ITS, tef1, and tub2 sequences were trimmed to a common length and concatenated using the Sequence Matrix software [30]. Concatenated sequences were aligned with MUSCLE [31] as implemented in MEGA7 [25], and edited manually for checking indels and single nucleotide polymorphisms. Phylogenetic analyses were performed in Mega with the maximum likelihood method using the Tamura–Nei model and 1000 bootstrap replications [32].

### 2.5. Pathogenicity Tests

The pathogenicity of an isolate of *Neofusicoccum parvum* (BOT 1D), sourced from lemon in Sicily, was assessed on twigs and stem of 2-year-old trees of three citrus varieties, *Citrus* × *limon* var. Monachello, *Citrus* × *limon* var. Femminello and citrange ‘Carrizo’ (*Citrus sinensis* Osbeck × *Poncirus trifoliata* Raf.), grown in a greenhouse maintained at 20 to 26 °C.

Two plants per variety were inoculated in three points, on two twigs and on stem per tree. A hole in each twig was made with a 3 mm cork-borer. For the inoculation, a 3 mm diam. mycelium plug from 5-d-old PDA culture of the test isolate (BOT 1D) was placed on the freshly wounded surface; the wound was covered with the excised bark disk and sealed with Parafilm^®^. The stem of each tree was inoculated 10 cm above the soil level (a single hole per stem) using the method above reported. Two trees per each citrus variety were inoculated with sterile agar (Controls).

The length and breadth of each resulting lesion were recorded 30 days after inoculation (d.a.i.), and the outer surface areas of the bark cankers on twigs and stems were calculated as ellipses.

Re-isolation was made from lesions and the identity of resulting fungal colonies was confirmed by both morphological and molecular analysis (ITS, tef1, and tub2 genes were sequenced) as described above.

## 3. Results

### 3.1. Symptoms

**Figure 2:**
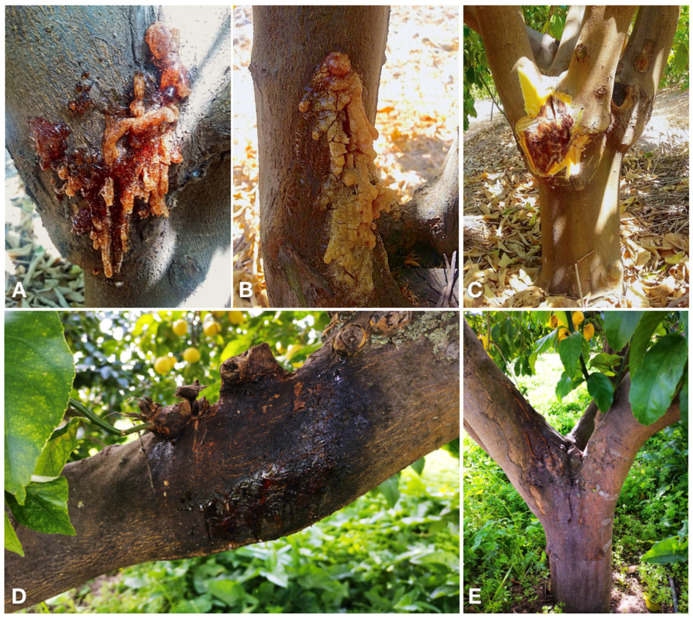
Symptoms of bot gummosis on lemon trees: (**A**,**B**) cankers with abundant gummous exudate on scaffold branches of ‘Femminello Siracusano 2Kr’ lemon (*Citrus* × *limon*) tree; (**C**) typical tan to brown discoloration of the wood beneath the bark of a canker; the discolored wood is visible after removing the bark; (**D**) gummous canker on a branch of lemon tree; (**E**) old canker with bark scaling at the level of main branch scaffolding, expanding upward to the secondary branches and downward to the grafting line between the scion and the rootstock.

**Figure 3:**
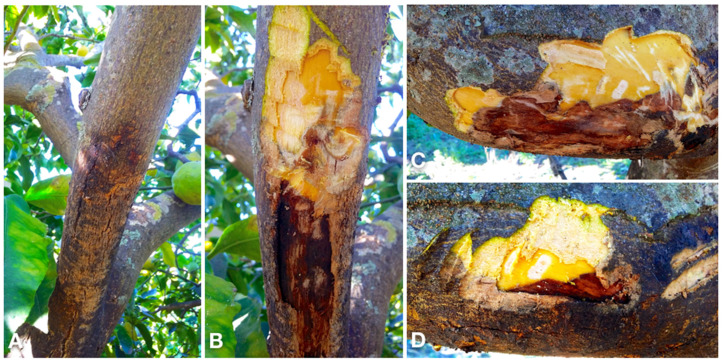
(**A**) Old canker on a secondary branch of lemon tree with longitudinal bark splitting at the base of the branch and the gum exudate in the advancing upward front, indicating the canker is still active; (**B**) the same as (**A**) after peeling the bark to show the dark brown discoloration of the wood beneath the bark; (**C**) typical chocolate brown discoloration of the wood underneath the bark; (**D**) a detail showing the necrotic bark of the canker exuding gum and the typical chocolate brown wood discoloration beneath the bark.

**Figure 4:**
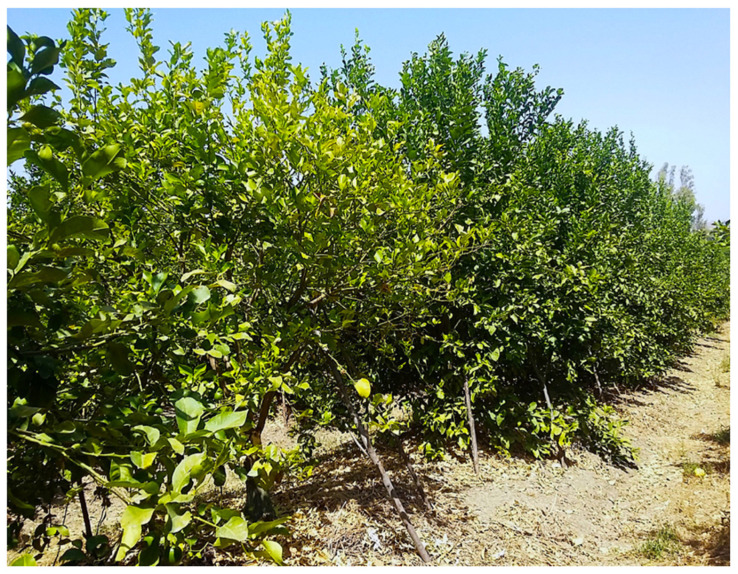
In the foreground, a lemon tree approximately 20-yr-old with yellowing of the canopy as a consequence of bot gummosis on scaffold branches.

Bot gummosis was detected on mature, fruit-bearing lemon trees both in commercial orchards and in trees planted singularly or in small groups in home gardens where trees were grown for both ornamental purposes and domestic consumption of fruits. Among the 25 sampled trees, one, in the province of Syracuse, was about 5-yr-old; five trees, of which three in two distinct sites of the province of Catania, one in the province of Messina and one in the province of Palermo were over 40-yr-old. The rest of the trees were between approximately 15 and 30 years old. The disease was common, but usually in commercial orchards symptomatic trees were scattered and the proportion of symptomatic trees in each orchard did not exceed 5%. Exceptionally, in an orchard in the municipality of Lentini (Syracuse) 30 out of 400, 20-yr-old trees were symptomatic. Typically, symptoms, i.e. cankers with an abundant gummous exudate, were observed on the main trunk and scaffold branches (Figure 2A–E) of trees. Another typical symptom was a chocolate to dark brown discoloration of the wood and the cambial face of the bark, which was visible after removing the bark (Figure 2C and Figure 3B–D). Cankers usually originated at the level of main branch scaffolding and progressively expanded upward to the secondary branches and downward to the grafting line between the scion and the rootstock (Figure 2A,C,D). However, in most cases they were restricted to the lemon scion and only exceptionally and in any case to a limited extent they expanded on sour orange (*Citrus* × *aurantium*) or citrange (*Citrus sinensis* × *Poncirus trifoliata*) rootstock. In a few cases, as a consequence of chronic infections the cankers girdled the trunk. Old cankers either ceased to produce gum or produced it only on the expanding edge; they showed longitudinal bark splittings on secondary branches and irregular cracking and scaling of the bark on trunk and scaffold branches (Figure 2E and Figure 3A,D). Leaf yellowing, defoliation, and dieback of single branches occurred commonly. Severely affected trees showed decline symptoms, including leaf chlorosis, severe crown thinning, and branch and twig blight and dieback (Figure 4).

### 3.2. Fungus Isolation and Morphological Identification

**Figure 5:**
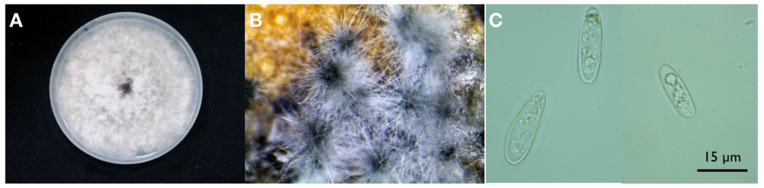
(**A**) Colony morphology of an isolate of *Neofusicoccum parvum* recovered from lemon on potato-dextrose-agar after 5 d incubation at 25 °C; (**B**) Pycnidia developed on MEA containing sterilized pine needles; (**C**) Unicellular, ellipsoidal, thin-walled hyaline conidia of *Neofusicoccum parvum* mounted in sterile distilled water.

A fungus with pale gray and rapidly growing colonies on potato-dextrose-agar (PDA) was constantly (from all samples and in all sampling sites) and consistently (around 100% of isolations) recovered from the inner bark with the typical brown discoloration. Based on the morphotype, i.e., colony morphology and conidial characteristics, all isolates were assigned to *Neofusicoccum*, a genus in the Botryosphaeriaceae family (Figure 5A,C). A set of 25 isolates, each from a distinct tree, was characterized further.

Colonies on MEA of all 17 isolates developed an abundant grayish aerial mycelium, which produced black pycnidia after two weeks of incubation at 25 °C (Figure 5B). On PDA mycelium was white and became smoky gray to gray-olivaceous after 5 d (Figure 4A). The mycelium was fast-growing and covered the 9 cm diam. Petri dishes after 5 d incubation at 25 °C in the dark. Optimum temperature for radial colony growth was between 25 and 30 °C for all the isolates tested (Table 3). Little growth was observed at 10 or 35 °C. Stromatic conidiomata were produced in pine needle cultures within 14 d. The conidiomata were globose and non-papillate to pyriform with a short, acute papilla, entire locule lined with conidiogenous cells, and measured 150–250 μm in diameter. Conidia were ellipsoidal with apex round and base flat, unicellular, and hyaline and measured 16.9–17.3 × 5.4–5.6 μm, with a mean length to width ratio = 3.2 (Figure 5C). Old conidia becoming 1–2-septate hyaline, or light brown with the middle cell darker than the terminal cells.

### 3.3. Molecular Identification

**Figure 6:**
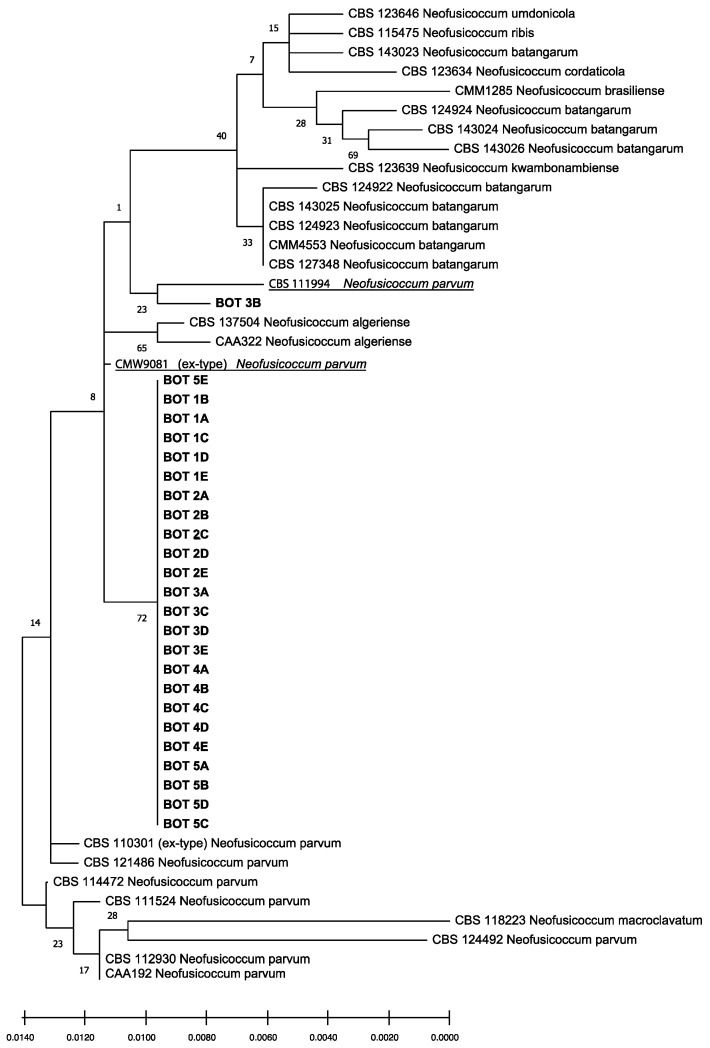
Phylogenetic tree of isolates of *Neofusicoccum parvum* sourced from lemon trees in this study (in bold), and reference isolates of *Neofusicoccum parvum* (underlined) and other closely related *Neofusicoccum* species and isolates (Table 1 and Table 2). The tree was built using concatenated sequences of ITS-5.8S-ITS2 region, tef1-α and β-tubulin genes. Numbers on nodes indicate the posterior probabilities from the maximum likelihood method.

The isolates obtained had identical ITS, tef1, and tub2 sequences. Preliminary BLAST analyses of these three gene regions yielded several identical sequences belonging to *Neofusicoccum* spp. but deposited with different taxa names. Consequently, this analysis enabled the identification at the genus level, but did not provide reliable information on the species. The phylogenetic analysis of the combined data set of sequences from ITS, tef1, and tub2 sequences (Figure 6) produced trees with a high concordance with those reported by Lopes et al. [27,29], Yang et al. [28], and Zhang et al. [7]. According to this analysis, isolates obtained from gummy cankers of *Citrus × limon* were identified as *Neofusicoccum parvum sensu stricto* (*s.s.*), since they clearly clustered with the ex-type (CMW9081 from *Populus nigra* [29]) and other reference isolates of this species (CBS 111994 from *Telopea* sp. [28]) and were differentiated from other *Neofusicoccum* isolates in the *Neofusicoccum* species complex [7].

### 3.4. Pathogenicity Tests

**Figure 7:**
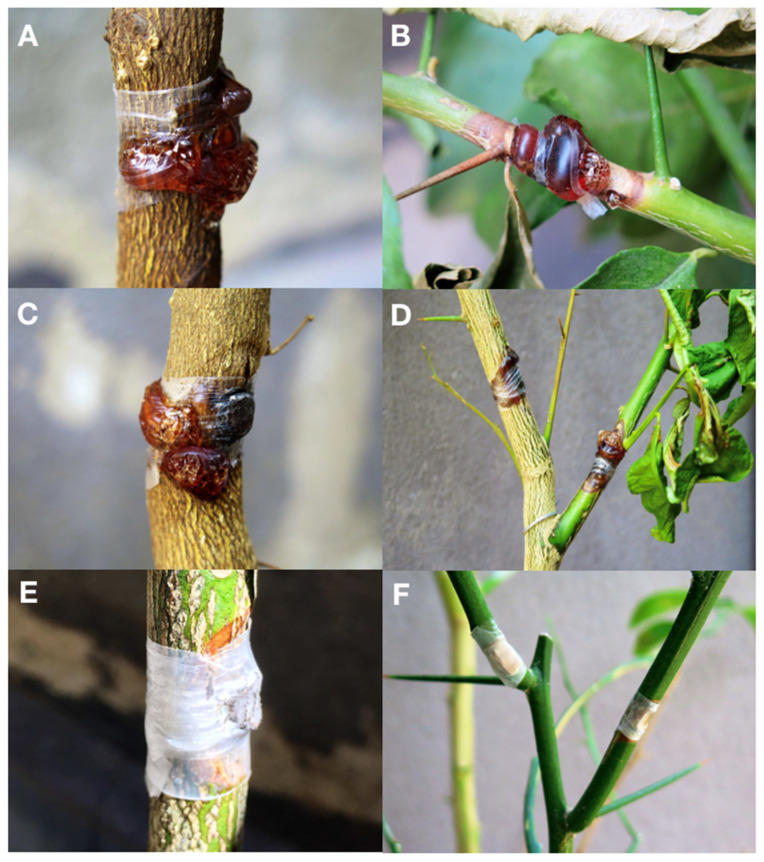
Gum exudate induced by wound-inoculation of *Neofusicoccum parvum* on stems and twigs of lemon ‘Femminello Siracusano 2kr’ (**A**,**B**) and ‘Monachello’ (**C**,**D**) 14 d a.i.; necrotic lesions without gummous flux on inoculated Citrange ‘Carrizo’ (**E**,**F**).

**Figure 8:**
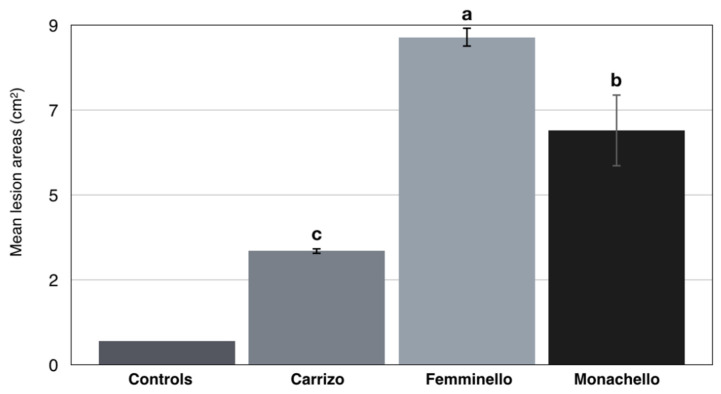
Mean lesion areas (cm^2^) on the stem of citrange (*Citrus sinensis × Poncirus trifoliata*) ‘Carrizo’ and lemon (*Citrus × limon*) cultivars ‘Femminello 2kr’ and ‘Monachello’, wound-inoculated with isolate BOT1D of *Neofusicoccum parvum*, 30 d.a.i. Controls (‘Femminello 2kr’ lemon, ‘Monachello’ lemon and ‘Carrizo’ citrange) stem inoculated with a sterile agar plug, showed no symptoms. Values sharing different letters are statistically different according to Tukey’s honestly significant difference, for *p* = 0.05.

In pathogenicity tests, *N. parvum* (isolate BOT 1D) induced gummy canker on twigs and stem of all three citrus varieties tested. Symptoms appeared at 14 d.a.i.; they were more severe on lemon cultivars ‘Femminello 2kr’(lesion areas ranging from 8.4 to 8.9 cm^2^ 30 d.a.i.), and ‘Monachello’ (lesion areas ranging from 6.5 to 6.8 cm^2^ 30 d.a.i.) and included abundant gummosis (Figure 7A–D), while gummosis was much less abundant on Citrange ‘Carrizo’ (lesion areas ranging from 3.1 to 3.3 cm^2^ 30 d.a.i.) (Figure 7E,F). Differences in mean lesion size between the three citrus varieties tested were significant, according to Tukey‘s HSD (Honestly Significant Difference) test (*p* < 0.05) (Figure 8). No symptoms were observed on the controls.

The *N. parvum* test isolate was re-isolated from inoculated plants and identified by sequencing and multi-loci phylogenetic analysis, while no fungal pathogens were isolated from control plants, thus fulfilling Koch’s postulates.

## 4. Discussion

Bot gummosis, traditionally regarded as a minor disease in citrus orchards [33], was found to be common and widespread in lemon groves of southern Italy. Although it is not as destructive as the malsecco disease caused by the mitosporic fungus *Plenodomus tracheiphilus* (syn. *Phoma tracheiphila*) [34], bot gummosis can be regarded as a major constraint for the lemon industry in this production area as it causes premature aging of trees and reduces their productivity. In this respect, the impact of bot gummosis in commercial lemon orchards is as severe as wood rot disease caused by species of Basidiomycetes and unlike wood rot, bot gummosis may affect trees under the age of 25 years [35]. The present study provided evidence that *Neofusicoccum parvum* in the family Botryosphaeriaceae is responsible for bot gummosis of lemon trees in Sicily and Calabria, the two major lemon producing Italian regions. This fungus was the only species of this family associated to the typical disease symptoms in all surveyed areas and the two most popular Italian lemon cultivars, ‘Femminello 2kr’ and ‘Monachello’, were shown to be very susceptible to the infections by this pathogen in pathogenicity assays. Consistently with symptoms observed on naturally infected trees in the field, pathogenicity tests revealed that *N. parvum* was very aggressive on ‘Femminello 2kr’ and ‘Monachello’, which reacted to the infection with the abundant production of gum exudate, while it was weakly virulent on ‘Carrizo’ citrange, commonly used as a citrus rootstock. These marked differences in susceptibility to bot gummosis among different citrus genotypes are in agreement with results of other Authors [10]. In pathogenicity trials carried out in Greece the citrumelo ‘Swingle’ (*Poncirus trifoliata* ×*C. paradisi*), commonly used as a rootstock, was proved to be the least susceptible to *N. parvum* and *D. foeniculina* infections among nine different citrus species [10]. *Neofusicoccum parvum* is a generalist, cosmopolitan pathogen, occurring in various environments with a temperate, Mediterranean, or subtropical climate. The host range of this species encompasses at least 90 botanical entities, especially woody angiosperms, including conifers and many horticultural plants [4,36,37,38,39,40,41,42,43,44]. In association with other fungal pathogens, including other Botryosphaeriaceae species, *N. parvum* is involved in the trunk disease complex of grapevine, also known as Botryosphaeria dieback, and is regarded as one of the most aggressive causal agent of this disease, which is responsible for severe economic losses in vineyards worldwide [45,46]. It was also reported as a pathogen of citrus from Australia, California, and Europe [8,47,48]. However, this is the first report of *N. parvum* as a pathogen of citrus in Italy.

In a previous study, it was shown that *N. parvum* is more aggressive than other Botryosphaeriaceae species associated to bot gummosis of citrus [3]. This may explain its prevalence on lemon, which is more susceptible to bot gummosis than other citrus species [49]. Like other species of *Neofusicoccum*, *N. parvum* produces phytotoxins, which probably act as virulence factors but none of them is host-specific [50,51,52]. Different *Neofusicoccum* species cause similar effects on plants, perhaps due to the production of these secondary metabolites. *Neofusicoccum parvum,* e.g., when inoculated on cactus pear cladodes was able to induce on this host the same symptoms as *N. batagarum* (13) and conversely in artificial inoculations *N. batangarum* induced on citrus plants the same symptoms as *N. parvum* [13]. Genes encoding for virulence factors that allow *N. parvum* to colonize the wood of the trunk and the transcriptional dynamics of grapevine genes during the stem colonization by this fungal pathogen have been investigated [53,54]. In general, the Botryosphaeriaceae, like other opportunistic albeit aggressive plant pathogens such as *Colletotrichum* spp., behave as entophytes, saprobes or latent pathogens shifting to an aggressive pathogenic life style when the host-plant is stressed [55,56,57,58,59]. It has been suggested that the widespread occurrence of *N. parvum* and related species is due to their polyphagy and cross-infection potential as well as to their behavior as endophytes or latent pathogens favoring a global spread through the movement of asymptomatic plants and plant propagation material [40,56,60,61]. According with this hypothesis, it can be speculated that the widespread occurrence of *N. parvum* in lemon growing areas of southern Italy may also depend on their close proximity with districts where the nursery production of citrus plants is concentrated. As a consequence, it can be assumed that most lemon trees in these areas have a common origin and *N. parvum* has been spread as a latent pathogen with nursery plants. This study provided evidence that restricted outbreaks of bot gummosis may occur in lemon groves of southern Italy. Local emergence probably due to environmental stresses is typical of diseases caused by Botryosphaeriaceae [13,42,62]. Extreme temperatures and water deficit are suspected to be the most frequent stressors favoring bot gummosis of citrus. These environmental factors condition not only the disease onset and development, but also the severity of symptom expression. A better understanding of the factors favoring the emergence of the bot gummosis in lemon orchards would be useful to adopt appropriate management strategies to prevent the disease.

## 5. Conclusions

The constant association between *Neofusicoccum parvum* and symptoms of bot gummosis on lemon trees and its aggressiveness towards two of the most popular Italian lemon cultivars (one traditional and one selected quite recently) clearly indicate the fungus is responsible for this disease in major lemon-growing areas of southern Italy. The occurrence of bot gummosis in a relatively vast geographical area where its presence has been documented since the past century, albeit with different common names, suggests this record of *N. parvum* on lemon is not occasional even if it is the first in Italy. Nursery plants are suspected to be the main vehicle for the wide distribution of this fungus whose life style as latent pathogen might have favored its spread through asymptomatic plants. It is noteworthy this study provided evidence of local outbreaks of bot gummosis triggered by environmental factors. The incidence and frequency of these outbreaks could be expected to increase in the future as a consequence of both climate change and the introduction of more susceptible lemon cultivars. With regard to the latter aspect, the marked difference in susceptibility shown by the two lemon cultivars in pathogenicity trials would suggest to extend the test to other lemon genotypes.

## Figures and Tables

**Table 1:** Identity of the *Neofusicoccum parvum* isolates sourced from gummy cankers of lemon (*Citrus* × *limon*) trees in southern Italy characterized in this study, and corresponding GenBank accession numbers.

Isolate	Species	Origin	Host, Variety	Accession Numbers		
				ITS	β-Tubulin	EF-1α
BOT 1A	*Neofusicoccum parvum*	Catania, Siciliy-Italy	*Citrus* ×*limon*	MW727244	MW789889	MW789904
BOT 1B	*N. parvum*	Catania, Siciliy-Italy	*C.* ×*limon*	MW727245	MW789890	MW789905
BOT 1C	*N. parvum*	Catania, Siciliy-Italy	*C.* ×*limon*	MW727246	MW789891	MW789906
BOT 1D	*N. parvum*	Catania, Siciliy-Italy	*C.* ×*limon*	MW727247	MW789892	MW789907
BOT 1E	*N. parvum*	Catania, Siciliy-Italy	*C.* ×*limon*	MW727248	MW789893	MW789908
BOT 2A	*N. parvum*	Syracuse, Siciliy-Italy	*C.* ×*limon*	MW727249	MW789894	MW789909
BOT 2B	*N. parvum*	Syracuse, Siciliy-Italy	*C.* ×*limon*	MW727250	MW789895	MW789910
BOT 2C	*N. parvum*	Syracuse, Siciliy-Italy	*C.* ×*limon*	MW727251	MW789896	MW789911
BOT 2D	*N. parvum*	Syracuse, Siciliy-Italy	*C.* ×*limon*	MW727252	MW789897	MW789912
BOT 2E	*N. parvum*	Syracuse, Siciliy-Italy	*C.* ×*limon*	MW727253	MW789898	MW789913
BOT 4A	*N. parvum*	Messina, Siciliy-Italy	*C.* ×*limon*	MW788562	MW789929	MW789919
BOT 4B	*N. parvum*	Messina, Siciliy-Italy	*C.* ×*limon*	MW788563	MW789930	MW789920
BOT 4C	*N. parvum*	Messina, Siciliy-Italy	*C.* ×*limon*	MW788564	MW789931	MW789921
BOT 4D	*N. parvum*	Messina, Siciliy-Italy	*C.* ×*limon*	MW788565	MW789932	MW789922
BOT 4E	*N. parvum*	Messina, Siciliy-Italy	*C.* ×*limon*	MW788566	MW789933	MW789923
BOT 5A	*N. parvum*	Palermo, Siciliy-Italy	*C.* ×*limon*	MW788567	MW789934	MW789924
BOT 5B	*N. parvum*	Palermo, Siciliy-Italy	*C.* ×*limon*	MW788568	MW789935	MW789925
BOT 5C	*N. parvum*	Palermo, Siciliy-Italy	*C.* ×*limon*	MW788569	MW789936	MW789926
BOT 5D	*N. parvum*	Palermo, Siciliy-Italy	*C.* ×*limon*	MW788570	MW789937	MW789927
BOT 5E	*N. parvum*	Palermo, Siciliy-Italy	*C.* ×*limon*	MW788571	MW789938	MW789928
BOT 3A	*N. parvum*	Reggio Calabria, Calabria-Italy	*C.* ×*limon*	MW727254	MW789899	MW789914
BOT 3B	*N. parvum*	Reggio Calabria, Calabria-Italy	*C.* ×*limon*	MW727255	MW789900	MW789915
BOT 3C	*N. parvum*	Reggio Calabria, Calabria-Italy	*C.* ×*limon*	MW727256	MW789901	MW789916
BOT 3D	*N. parvum*	Reggio Calabria, Calabria-Italy	*C.* ×*limon*	MW727257	MW789902	MW789917
BOT 3E	*N. parvum*	Reggio Calabria, Calabria-Italy	*C.* ×*limon*	MW727258	MW789903	MW789918

**Table 2:** GenBank accession numbers of sequences of *Neofusicoccum* spp. isolates of different geographical and host origins used as reference in phylogenetic analyses.

Species	Isolate	Origin	Host	Source	GenBank Accession Number		
					ITS	tef1	β-Tubulin
*N. algeriense*	CAA 322	Portugal	*Malus domestica*	[29]	KX505906	KX505894	KX505916
*N. algeriense*	CBS 137504	Algeria	*Vitis vinifera*	[29]	KJ657702	KX505893	KX505915
*N. batangarum*	CBS 124922	Cameroon	*Terminalia catappa*	[29]	FJ900606	FJ900652	FJ900633
*N. batangarum*	CBS 143023	Italy, Favignana	*Opuntia ficus-indica*	[13]	MF414730	MF414768	MF414749
*N. batangarum*	CBS 143025	Italy, Linosa	*Opuntia ficus-indica*	[13]	MF414747	MF414785	MF414766
*N. batangarum*	CBS 127348	USA, Florida	*Schinus terebinthifolius*	[28]	HM357636	KX464674	KX464952
*N. batangarum*	CMM4553	Brasil	*Anacardium* sp.	Unpublished	KT728917	KT728921	KT728913
*N. batangarum*	CBS 124923	Cameroon	*Terminalia catappa*	[29]	FJ900608	FJ900654	FJ900635
*N. batangarum*	CBS 124924	Cameroon	*Terminalia catappa*	[29]	FJ900607	FJ900653	FJ900634
*N. batangarum*	CBS 143026	Italy, Lampedusa	*Opuntia ficus-indica*	[13]	MF414748	MF414786	MF414767
*N. batangarum*	CBS 143024	Italy, Ustica	*Opuntia ficus-indica*	[13]	MF414738	MF414776	MF414757
*N. brasiliense*	CMM1285	Brazil	*Mangifera indica*	[29]	JX513628	JX513608	KC794030
*N. cordaticola*	CBS 123634	South Africa	*Syzygium cordatum*	[29]	EU821898	EU821868	EU821838
*N. kwambonambiense*	CBS 123639	South Africa	*Syzygium cordatum*	[29]	EU821900	EU821870	EU821840
*N. macroclavatum*	CBS 118223	Australia	*Eucalyptus globulus*	[29]	DQ093196	DQ093217	DQ093206
*N. parvum*	CBS 111994	Australia	*Telopea* sp.	[28]	AF452519	KX464702	KX464982
*N. parvum*	CAA 192	Portugal	*Ferula communis*	[29]	KX505905	KX505892	KX505913
*N. parvum*	CBS 112930	South Africa	*Vitis vinifera*	[28]	AY343467	AY343359	KX464983
*N. parvum*	CBS 121486	Spain	*Vitis vinifera* cv. Parellada	[28]	EU650672	KX464707	KX464992
*N. parvum*	CBS 114472	USA	*Leucadendron* sp.	[28]	AF452523	FJ150710	KX464987
*N. parvum*	CBS 111524	USA, Hawaii	*Protea cynaroides*	[28]	AF452524	FJ150709	KX465009
*N. parvum*	CBS 124492	Zambia	*Syzygium guineense*	[28]	FJ655000	KX464684	KX464962
*N. parvum*	CBS 110301(ex-type)	Portugal	*Vitis vinifera*	[29]	AY259098	AY573221	EU673095
*N. parvum*	CMW9081 (ex-type)	New Zealand	*Populus nigra*	[29]	AY236943	AY236888	AY236917
*N. ribis*	CBS 115475	USA	*Ribes* sp.	[29]	AY236935	AY236877	AY236906
*N. umdonicola*	CBS 123646	South Africa	*Syzygium cordatum*	[29]	EU821905	EU821875	EU821845

**Table 3:** Mean radial growth rates of colonies of representative *Neofusicoccum parvum* isolates of different geographical origin on PDA at three different temperatures, as determined after 3 d of incubation.

Isolates of *N. parvum*	15 °C (mm d-1) ± S.D. ^a^	25 °C (mm d-1) ± S.D. ^a^	30 °C (mm d-1) ± S.D. ^a^
BOT 1A	3.73 ± 0.07	7.90 ± 1.08	6.71 ± 0.27
BOT 1D	3.11 ± 0.91	7.73 ± 0.28	7.38 ± 0.47
BOT 2A	3.41 ± 0.05	7.55 ± 0.32	7.06 ± 0.28
BOT 2D	3.53 ± 0.62	6.68 ± 0.90	5.58 ± 0.28
BOT 3A	3.45 ± 0.39	7.06 ± 0.28	6.23 ± 0.20
BOT 3D	3.30 ± 0.17	6.83 ± 0.32	6.63 ± 0.46
BOT 4A	3.42 ± 0.06	6.58 ± 0.62	7.38 ± 0.47
BOT 5D	3.28± 0.17	7.22 ± 0.42	5.37 ± 0.38

^a^ Mean of four replicate Petri dishes.
